# Optimal sampling of MRI slices for the assessment of knee cartilage volume for cross-sectional and longitudinal studies

**DOI:** 10.1186/1471-2474-6-10

**Published:** 2005-02-20

**Authors:** Guangju Zhai, Changhai Ding, Flavia Cicuttini, Graeme Jones

**Affiliations:** 1 Menzies Research Institute, University of Tasmania, Hobart, Australia; 2 Department of Epidemiology and Preventive Medicine, Monash University, Alfred Hospital, Prahran, Vic, Australia

## Abstract

**Background:**

MRI slices of 1.5 mm thickness have been used in both cross sectional and longitudinal studies of osteoarthritis, but is difficult to apply to large studies as most techniques used in measuring knee cartilage volumes require substantial post-image processing. The aim of this study was to determine the optimal sampling of 1.5 mm thick slices of MRI scans to estimate knee cartilage volume in males and females for cross-sectional and longitudinal studies.

**Methods:**

A total of 150 subjects had a sagittal T1-weighted fat-suppressed MRI scan of the right knee at a partition thickness of 1.5 mm to determine their cartilage volume. Fifty subjects had both baseline and 2-year follow up MRI scans. Lateral, medial tibial and patellar cartilage volumes were calculated with different samples from 1.5 mm thick slices by extracting one in two, one in three, and one in four to compare to cartilage volume and its rate of change. Agreement was assessed by means of intraclass correlation coefficient (ICC) and Bland & Altman plots.

**Results:**

Compared to the whole sample of 1.5 mm thick slices, measuring every second to fourth slice led to very little under or over estimation in cartilage volume and its annual change. At all sites and subgroups, measuring every second slice had less than 1% mean difference in cartilage volume and its annual rate of change with all ICCs ≥ 0.98.

**Conclusion:**

Sampling alternate 1.5 mm thick MRI slices is sufficient for knee cartilage volume measurement in cross-sectional and longitudinal epidemiological studies with little increase in measurement error. This approach will lead to a substantial decrease in post-scan processing time.

## Background

Osteoarthritis (OA) is the most common form of arthritis and a leading cause of musculoskeletal disability in most developed countries [1]. The knee is one of the most frequently affected joints with a prevalence of 30% in people older than 65 years [2] and high resultant disability [3]. Defects in cartilage are widely considered to be the initial problem in OA [4,5], although this viewpoint is not shared by all investigators [6]. Detection of cartilage morphological change is critical in the evaluation, diagnosis, and monitoring of OA. Conventional radiography is used in evaluating the progression of OA but is limited by its inability to directly visualise cartilage. Magnetic resonance imaging (MRI) offers the distinct advantage of detecting morphologic changes in articular cartilage and is a sensitive and accurate test for evaluating articular cartilage non-invasively [7-11]. The correlation coefficient is 0.99 between knee cartilage volumes measured by MRI and the true volumes by means of water displacement [9]. This method uses 1.5 mm thick MRI slices and has high reproducibility with coefficients of variation of 2–3% [12] and has been used in both cross sectional and longitudinal studies of OA [12-15]. However, the method is difficult to apply to large studies as most techniques used in measuring knee cartilage volumes require substantial post-image processing [12] and the process has not yet been automated. One possible solution is to select a sample from within the 1.5 mm thick slices to reduce the post-image processing time, as has been reported for the estimation of brain compartment volume [16] and fetal volume[17]. The aim of the study, therefore, was to determine the optimal sampling of 1.5 mm thick MRI slices required to estimate the volumes of and rate of change in lateral, medial tibial and patellar cartilage with minimal increase in measurement error.

## Methods

### Subjects

The present study consisted of two datasets; one was part of the Tasmanian Older Adult Cohort Study (TASOAC), an ongoing prospective population-based study aimed at identifying the environmental, genetic, and biochemical factors associated with the development and progression of OA at multiple sites (hand, knee, hip, and spine), which commenced in 2002. Subjects aged between 50 and 79 years were selected randomly from the electoral roll of Southern Tasmania, with an equal number of males and females. Another dataset was a younger adult sample from the Knee Cartilage Volume study (KCV) as previously reported [15]. Both studies were approved by the Southern Tasmanian Health and Medical Human Research Ethics Committee and all subjects provided informed written consent.

### MRI

An MRI scan of the right knee was performed on all subjects. Knee cartilage volume was determined by means of image processing on an independent work station using the software program Osiris as previously described [12,15]. Two observers were utilised. Knees were imaged in the sagittal plane on a 1.5-T whole body magnetic resonance unit (Picker) with use of a commercial transmit-receive extremity coil. The same machine scanned all knees and Philips Quality Procedure (Philips ACR Support Program, XJR153-2922.03) was utilised for MRI slice thickness quality assurance. The following image sequence was used: a T1-weighted fat saturation 3D gradient recall acquisition in the steady state; flip angle 55 degrees; repetition time 58 msecs; echo time 12 msec; field of view 16 cm; 60 partitions; 512 × 512 matrix; acquisition time 11 min 56 sec; one acquisition. Sagittal images were obtained at a partition thickness of 1.5 mm and an in-plane resolution of 0.31 × 0.31 (512 × 512 pixels). The image data were transferred to the workstation. The volumes of individual cartilage plates (medial tibial, lateral tibial and patella) were isolated from the total volume by manually drawing disarticulation contours around the cartilage boundaries on a slice-by-slice basis. All individual slice areas for each cartilage site and each subject were subsequently transferred to and recorded on a spreadsheet. The total area of each individual cartilage was then multiplied by the slice thickness to produce a volume estimate. This "all slice" estimate of cartilage volume (based on slice thickness of 1.5 mm) was used as the gold standard for other comparisons.

Then, the volumes of all individual cartilage plates were recalculated based on different sampling intervals from 1.5 mm thick slices by extracting one in two, one in three, and one in four slice areas from the individual data file. These were then summed and the total was multiplied by the corresponding slice distance.

Femoral cartilage volume was not assessed in this study as it is strongly correlated with tibial cartilage volume and thus adds little extra information [18], tibial cartilage volume is the parameter that is most frequently examined in the literature [12,19-23], and femoral cartilage volume has worse reproducibility than tibial cartilage volume [11].

### Other measurements

Weight was measured to the nearest 0.1 kg (with shoes, socks and bulky clothing removed) using a single pair of electronic scales (Seca Delta Model 707) which were calibrated using a known weight at the beginning of each clinic. Height was measured to the nearest 0.1 cm (with shoes and socks removed) using a stadiometer. Body Mass Index (BMI) was calculated as weight (kg) / height (m^2^).

A standing AP semi-flexed view of the right knee was performed in all subjects. Radiographs were then assessed utilising the Altman atlas[24]. Each of the following was assessed: medial joint space narrowing (0–3), lateral joint space narrowing (0–3), medial osteophytes (femoral and tibial combined) (0–3) and lateral osteophytes (femoral and tibial combined) (0–3). Each score was arrived at by consensus with two readers simultaneously assessing the radiograph with immediate reference to the atlas. Any knee ROA was defined as total score ≥ 1. The total score could vary from 0–12. This method had high reproducibility in our hands with ICCs >0.98 [25].

### Statistics

Descriptive statistics of the characteristics of the study subjects were tabulated. The annual change in knee cartilage volume was calculated as percent change by means of dividing absolute volume change by baseline cartilage volume. Intraclass correlation coefficient was utilized to assess the measurement agreement. The difference in cartilage volume measured with different samples extracting one in two, one in three, and one in four 1.5 mm thick slices of MR image compared to that measured using 1.5 mm thickness was calculated and expressed as percent absolute difference. Desirable agreement was defined as an ICC ≥ 0.98 with ≤ 1% difference between two measurements. In addition, Bland & Altman plots [26] were also utilized. Desirable agreement was defined as the mean difference between two measurements close to zero with 95% of individual differences being within 2 SD. All analyses were performed using the SPSS statistical package (version 12.1, SPSS, Chicago, IL).

## Results

A total of 150 subjects took part in this study: 100 subjects with cross-sectional data (female: 48, male: 52) were from the TASOAC study and 50 subjects with longitudinal data (female: 31, male: 19) were from the KCV study. Characteristics of the study sample are presented in Table 1. Subjects from TASOAC were older, heavier and had a higher prevalence of ROA than those from KCV. Most of participants with ROA were mild with a total ROA score ≤ 3 out of 12. Lateral and medial tibial cartilage volumes were lower in subjects from KCV than those from TASOAC.

**Table 1:** Characteristics of the study population*

	TASOAC dataset N = 100	KCV dataset N = 50
Age (year)	62.3(7.6)	42.8(6.1)
Sex (female %)†	48	62
Height (cm)	167.4(8.7)	168.6(7.9)
Weight (kg)	76.0(15.0)	73.9(13.7)
BMI (kg/m^2^)	27.1(4.3)	25.9(4.1)
Any knee ROA (%)†	62	18
Knee ROA total score (0-**12**)	1.3 (1.7)	0.2(0.7)
Lateral tibial cartilage volume (ml)‡	3.0(0.7)	2.6(0.5)
Medial tibial cartilage volume (ml)‡	2.7(0.5)	2.2(0.5)
Patellar tibial cartilage volume (ml)‡	3.5(1.0)	3.5(0.9)
Lateral tibial cartilage volume change (%) per year‡	-	-1.2(3.4)
Medial tibial cartilage volume change (%) per year‡	-	-2.9(3.9)
Patellar cartilage volume change (%) per year‡	-	-3.8(3.4)

*Values are mean (SD) except for indicated. BMI: body mass index. ROA: radiographic osteoarthritis. † Percentage. ‡ Measured with the whole sample of 1.5 mm thick MRI slices.

**Figure 1:**
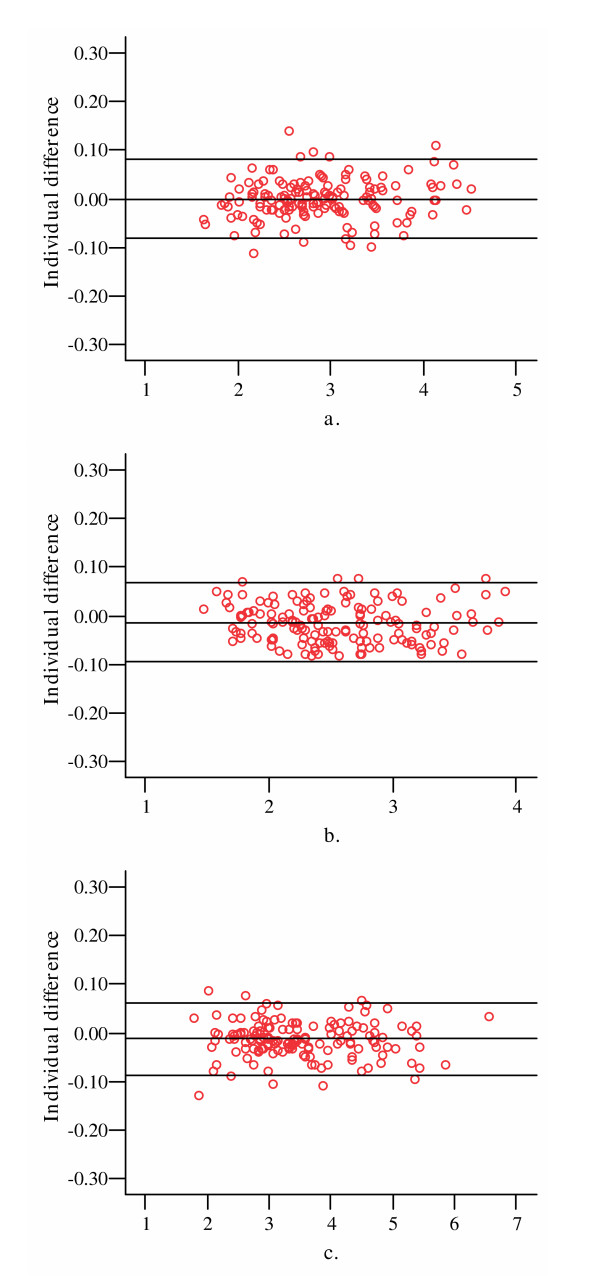
Bland & Altman plots of cartilage volume measured by every second 1.5 mm thick MRI slice compared to that measured by the total sample at lateral (a), medial tibial (b), and patellar (c) sites. The x-axis represents average values of two measurements while the y-axis represents the individual difference between two measurements, and the three horizontal lines stand for mean individual difference ± 2 SD.

In cross-sectional analysis, compared to the cartilage volume measured using 1.5 mm thickness, decreasing the number of the slices by extracting one in two to one in four led to a very little underestimation in the magnitude of the average cartilage volume at lateral, medial tibial and patellar sites with ICCs of 0.98–1.00 (Table 2). The maximum underestimation was 3.3% at the medial tibial site with one in four slices (Table 2). Similar results were obtained when the analysis was done separately for people with and without ROA (Table 3) although the differences tended to be larger in the ROA group. The difference also tended to be larger for medial tibial cartilage in the TASOAC sample and lateral tibial cartilage for the KCV sample (Table 2). At all sites and subgroups, cartilage volume measured with one in two slices had less than 1% difference compared to that measured with all 1.5 mm slices with an ICC of 1.0 (Table 2 &3). Bland & Altman plots showed that the mean difference was zero for lateral tibial cartilage and -0.01 ml for medial tibial and patellar cartilage with 95% of individual differences within ± 2SD. The variability was random and uniform throughout the range of cartilage volume (Figure 1).

**Table 2:** Agreement analysis of knee cartilage volume measured with different samples of 1.5 mm thick MRI slices*

	Whole sample (n = 150)		TASOAC sample (n = 100)		KCV sample (n = 50)	
	%Difference (SD)	ICC†	%Difference (SD)	ICC†	%Difference (SD)	ICC†

Lateral tibial cartilage						
*The whole sample*	Reference	Reference	Reference	Reference	Reference	Reference
*1/2 whole sample‡*	-0.04(1.5)	1.00	0.35(1.4)	1.00	-0.84(1.4)	1.00
*1/3 whole sample‡*	-0.61(2.3)	1.00	0.11(2.1)	1.00	-2.09(1.8)	1.00
*1/4 whole sample‡*	-1.12(3.4)	1.00	-0.11(3.0)	1.00	-3.18(3.3)	0.99
Medial tibial cartilage						
*The whole sample*	Reference	Reference	Reference	Reference	Reference	Reference
*1/2 whole sample‡*	-0.50(1.7)	1.00	-0.98(1.4)	1.00	0.46(1.7)	1.00
*1/3 whole sample‡*	-1.70(3.3)	0.99	-2.97(2.8)	0.99	0.83(2.9)	1.00
*1/4 whole sample‡*	-3.27(5.0)	0.98	-5.09(3.9)	0.97	0.38(4.9)	0.99
Patellar cartilage						
*The whole sample*	Reference	Reference	Reference	Reference	Reference	Reference
*1/2 whole sample‡*	-0.36(1.2)	1.00	-0.40(1.2)	1.00	-0.29(1.3)	1.00
*1/3 whole sample‡*	-0.91(2.0)	1.00	-0.93(2.0)	1.00	-0.86(1.9)	1.00
*1/4 whole sample‡*	-2.24(3.0)	1.00	-2.12(2.9)	1.00	-2.50(3.3)	1.00

* SD: standard deviation. ICC: intraclass correlation coefficient. † All P < 0.001. ‡ Derived by extracting one in two, one in three, or one in four of the 1.5 mm thick MRI slices.

**Table 3:** Agreement analysis of cartilage volume measured with different samples of 1.5 mm thick MRI slices in people with and without ROA*

	ROA absent (n = 76)		ROA present (n = 68)	
	Difference (SD)	ICC†	Difference (SD)	ICC†

Lateral tibial cartilage				
*The whole sample*	Reference	Reference	Reference	Reference
*1/2 whole sample‡*	-0.30(1.4)	1.00	0.24(1.6)	1.00
*1/3 whole sample‡*	-1.14(2.3)	1.00	-0.01(2.1)	1.00
*1/4 whole sample‡*	-1.85(3.4)	0.99	-0.29(3.4)	1.00
Medial tibial cartilage				
*The whole sample*	Reference	Reference	Reference	Reference
*1/2 whole sample‡*	-0.39(1.7)	1.00	-0.77(2.2)	1.00
*1/3 whole sample‡*	-1.20(3.3)	0.99	-2.13(3.4)	0.99
*1/4 whole sample‡*	-2.56(5.3)	0.98	-3.77(4.5)	0.98
Patellar cartilage				
*The whole sample*	Reference	Reference	Reference	Reference
*1/2 whole sample‡*	-0.38(1.2)	1.00	-0.40(1.2)	1.00
*1/3 whole sample‡*	-0.87(1.9)	1.00	-1.10(2.0)	1.00
*1/4 whole sample‡*	-2.02(2.9)	1.00	-2.50(3.2)	1.00

*Six subjects had missing values for ROA. Difference in cartilage volume measured with different thick slices of MR images is expressed as percentage. ICC: intraclass correlation coefficient. ROA: radiographic osteoarthritis. SD: standard deviation. † All P < 0.001. ‡ Derived by extracting one in two, one in three, or one in four 1.5 mm thick MRI slices.

**Figure 2:**
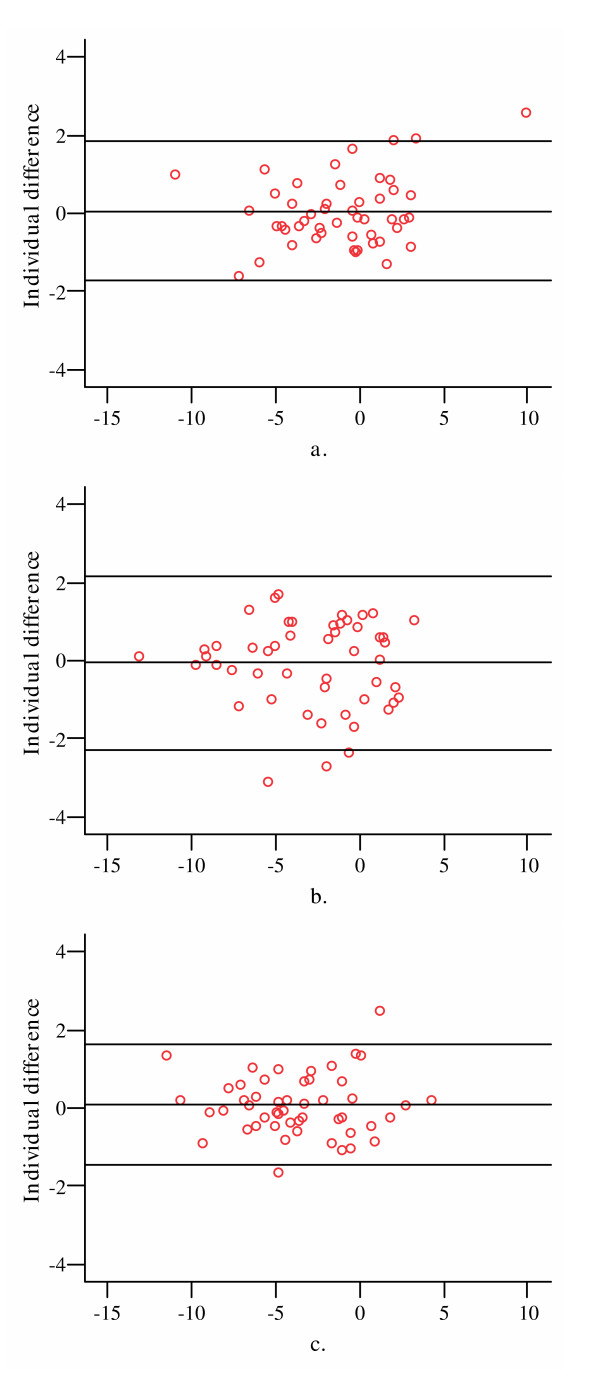
Bland & Altman plots of the annual change in cartilage volume measured by every second 1.5 mm thick MRI slice compared to that measured by the total sample at lateral (a), medial tibial (b), and patellar (c) sites. The annual change in cartilage volume was expressed as a percentage. The x-axis represents average values of two measurements while the y-axis represents the individual difference between two measurements, and the three horizontal lines stand for mean individual difference ± 2 SD.

Similarly, in longitudinal analysis, compared to the cartilage volume change using 1.5 mm thick slices, decreasing the number of the slices by extracting one in two to one in four slices led to very little over or under estimation of the mean changes in cartilage volume at lateral, medial tibial and patellar sites (Table 4). The mean difference ranged from -0.05% to 0.14% with the maximum difference at the patellar site. ICCs ranged from 0.85 to 0.99 (Table 4). The difference became larger but all were ≤ 1% in subjects with and without ROA (Table 4). At all sites, the annual change in cartilage volume measured with one in two slices had an ICC ≥ 0.98 with less than 0.3% difference compared to that measured using all the slices. Bland & Altman plots showed that 95% of the individual differences were within ± 2 SD and the variability was random and uniform throughout the range of cartilage volume (Figure 2).

**Table 4:** Agreement analysis of the annual change in knee cartilage volume measured with different samples of 1.5 mm thick MRI slices*

	Whole sample (n = 50)		ROA present (n = 9)		ROA absent (n = 41)	
	Difference (SD)	ICC†	Difference (SD)	ICC†	Difference (SD)	ICC†

Lateral tibial cartilage						
*The whole sample*	Reference	Reference	Reference	Reference	Reference	Reference
*1/2 whole sample‡*	0.06(0.9)	0.99	0.23(1.1)	0.99	0.02(0.9)	0.98
*1/3 whole sample‡*	0.05(1.5)	0.96	-0.65(1.4)	0.98	0.20(1.5)	0.95
*1/4 whole sample‡*	-0.03(2.2)	0.92	-0.04(2.4)	0.95	-0.02(2.2)	0.91
Medial tibial cartilage						
*The whole sample*	Reference	Reference	Reference	Reference	Reference	Reference
*1/2 whole sample‡*	-0.05(1.1)	0.98	-0.29(1.0)	0.99	0.00(1.1)	0.98
*1/3 whole sample‡*	-0.03(1.8)	0.95	0.24(1.8)	0.97	-0.10(1.8)	0.95
*1/4 whole sample‡*	0.02(3.0)	0.85	-1.04(2.7)	0.92	0.25(3.1)	0.83
Patellar cartilage						
*The whole sample*	Reference	Reference	Reference	Reference	Reference	Reference
*1/2 whole sample‡*	0.10(0.8)	0.99	-0.07(0.7)	1.00	0.13(0.8)	0.99
*1/3 whole sample‡*	0.10(1.5)	0.96	-0.18(1.4)	0.98	0.16(1.5)	0.95
*1/4 whole sample‡*	0.14(1.8)	0.93	0.61(1.5)	0.97	0.03(1.9)	0.92

* Difference in the annual change in cartilage volume was expressed in percentage. SD: standard deviation. ROA: radiographic osteoarthritis. ICC: intraclass correlation coefficient. † All P < 0.001. ‡ Derived by extracting one in two, one in three, or one in four 1.5 mm thick MRI slices.

## Discussion

This study suggests that lateral, medial tibial and patellar cartilage volumes measured from up to one in four 1.5 mm thick slices are quite comparable to those obtained from 1.5 mm thick slices. If the agreement is defined at high levels expected to lead to minimal measurement error, then knee cartilage volume can be measured sufficiently and accurately with one in two slices both cross-sectionally and longitudinally regardless of ROA status and./or reader. This approach will lead to a substantial decrease in post-scan processing time and make large-scale studies of knee cartilage volume more feasible.

Currently, there is no reported information on the number of the slices of MRI scans to measure cartilage volume apart from a recent paper from our own group which had similar findings to this study with different readers and geographic location [27]. In a study estimating fetal volume by MRI, Roberts et al reported that using the same thickness of MRI slices (10 mm), volume measured from the low sampling intensity (the distance between scan section midplanes T = 4.5 cm) was virtually identical to those obtained with the high sampling intensity (T = 1.5 cm) with a coefficient of error (CE) < 5% [17]. In the study estimating brain compartment volume from MR Cavalieri slices [16], irrespective of slice thickness, a minimum of 3, 5, and 10 slices provided estimates of the true total volume of grey matter and white matter in the cerebrum with coefficients of error (CEs) of 10, 5, and 3%. For a given number of slices CE decreases rapidly when the slices are thicker than the gaps between them; when the slices are thinner than the gaps, then CE is similar to that in the situation when the slice thickness is zero. The current study demonstrates similar results for knee cartilage. Decreasing the number of slices by extracting up to one in four 1.5 mm slices resulted in a very little underestimation in average volume of lateral, medial tibial and patellar cartilage. The maximum mean difference in cartilage volume obtained from one in four slices to that obtained from all slices was 3.3%, which is substantially smaller than the difference of 9% between cartilage volume obtained from 1.5 mm thick slices of MR image and that measured by means of water displacement [9,19,28,29]. The difference increased slightly when we analysed the data separately for people with and without ROA, but the results were similar for both groups, suggesting ROA within the range we report has very limited effect on the cartilage volume measured with subsamples of MRI slices. If we arbitrarily define an ICC ≥ 0.98 with ≤ 1% difference as optimal as it is expected to minimise the measurement error and only slightly increase the variance, then cartilage volume and its rate of change can be measured accurately with one in two 1.5 mm thick slices for lateral, medial tibial and patellar cartilage. Bland & Altman plots confirmed this with a random scatter about zero as would be expected if there is no difference between two measurements and uniform variability throughout the range of measurements. Of note, for longitudinal data even decreasing the number of slices by extracting up to one in four resulted in a maximum difference of 0.14% in mean annual change in cartilage volume which is very small when compared to the 5% cartilage loss annually we have reported in patients with OA [30]. Thus, a subsample of MRI slices could also be utilised with marked decreases in processing time allowing greater numbers of subjects to be studied offsetting the accompanying increase in measurement error.

Ideally, the more slices used, the more accurate the estimation of the object's volume, as they may contain more information. However, for a completely regular structure, such as a cylinder, the area of a single slice with length gives an exact volume. It is therefore reassuring but not surprising that the current study demonstrates a minimum reduction in the knee cartilage volume and volume change over time as tibial and patellar cartilages have a relatively regular structure. A different interpretation may apply to femoral cartilage and we do not have data on this imaging site.

The current study simply examined the effect of decreasing the number of slices on the estimation of knee cartilage volume and volume change while all other variables were kept constant. We did not re-scan the study subjects but simply estimated the cartilage volume by using one in two, one in three, or one in four slices. This has an advantage of allowing us to examine the single effect of sampling intensity in the situation where all other variables such as re-positioning the subject and measurement were kept constant. The effect of these errors on measurement have been well-documented [9,31]. For longitudinal analysis, all the MR images were processed by a single observer. For cross sectional analysis, two observers processed the MR images, one for TASOAC data, and another for the KCV study. However, the difference was even smaller in the whole sample than in the two separate samples providing reassurance that our results may be generalisable to different observers as documented with different readers and machines in Melbourne [27].

The current study has a number of potential limitations. Firstly, which sampling intensity should be used in the MRI scan of knee cartilage depends on the purpose of the measurement. Our results cannot be applied to individual cartilage volume, but only for mean cartilage volume in groups as the individual difference in cartilage volume increases with decreasing sampling intensity. Secondly, decreasing sampling intensity will increase measurement error as the remaining slices focus on different portions of the irregularly shaped cartilage. Depending on what particular surfaces remain, however, the overall volume may be increased or decreased. If this is random, then the mean will remain the same as demonstrated in the current study. Thirdly, the ICC can be influenced by traits in the sample in which it is assessed. Age, sex and BMI have been reported to be associated with knee cartilage volume [32]. These may result in a higher ICC in the current study, as between-subject variance would become larger. However, subgroup analyses by sex, BMI (< 25, >= 25), and age (<50, >= 50 yr) did not change the results (data not shown). Further analysis using the Bland & Altman method confirmed the good agreement and interchangability between thick and thin slices, indicating that the result of the current study should be applicable to other populations regardless of the demographic factors related to cartilage volume. Fourthly, the participants in the study had only mild ROA, and these conclusions may not apply to subjects with more advanced OA. Lastly, the annual change in cartilage volume in our sample can not be generalized to other populations as half of our longitudinal study sample had a higher genetic susceptibility to OA [23,33].

## Conclusion

Knee cartilage volume and its rate of change can be accurately measured with every second 1.5 mm thick MR slice. This approach will lead to a substantial decrease in post-scan processing time and make large-scale studies of knee cartilage volume more feasible.

## Competing interests

The author(s) declare that they have no competing interests.

## Authors' contributions

GZ designed and carried out the study planning, data collection, analysis and interpretation of the analysis, and preparation of the manuscript. CD participated in data collection and critical revision of the manuscript. FC participated in the study planning and critical revision of the manuscript. GJ designed the study, participated in analysis and interpretation of the analysis, and critical revision of the manuscript. All authors read and approved the final manuscript.

## Pre-publication history

The pre-publication history for this paper can be accessed here:


